# MetaboCraft: building a Minecraft plugin for metabolomics

**DOI:** 10.1093/bioinformatics/bty102

**Published:** 2018-03-28

**Authors:** Anargyros Megalios, Rónán Daly, Karl Burgess

**Affiliations:** 1 Glasgow Polyomics, University of Glasgow, Glasgow, UK; 2 Institute of Infection, Immunity and Inflammation, College of Medical Veterinary and Life Sciences, University of Glasgow, Glasgow, UK

## Abstract

**Motivation:**

The rapid advances in metabolomics pose a significant challenge in presentation and interpretation of results. Development of new, engaging visual aids is crucial to advancing our understanding of new findings.

**Results:**

We have developed MetaboCraft, a Minecraft plugin which creates immersive visualizations of metabolic networks and pathways in a 3D environment and allows the results of user experiments to be viewed in this context, presenting a novel approach to exploring the metabolome.

**Availability and implementation:**

https://github.com/argymeg/MetaboCraft/; https://hub.docker.com/r/ronandaly/metabocraft/

**Supplementary information:**

Supplementary data are available at *Bioinformatics* online.

## 1 Introduction

Metabolomics as a field has expanded rapidly in recent years, owing both to increased interest as well as advances in instrument throughput and resolution, accordingly resulting in the accumulation of a vast metabolomic data repository (Haug *et al.*, 2017), presenting ever-increasing challenges to scientists attempting to interpret this data. The utility of visualizations in aiding interpretation of scientific datasets is well described (Auber, 2004), however the advances in the development of visual aids for metabolomics have been slower, mostly centring on cosmetic refinements to the traditional, 2D, metabolic map paradigm.

PiMP (Gloaguen *et al.*, 2017) is a metabolomics pipeline currently in development, which aims to streamline and standardize the process of metabolomics analysis into one tool. Exploring new and innovative approaches to data visualization is an integral part of the development of PiMP.

At a conceptual level, metabolic maps can be interpreted as mathematical graph objects, enabling the application of widely applied methods towards both their design and their interpretation (Cottret and Jourdan, 2010). MetExplore (Cottret *et al.*, 2010) is a web service and repository of metabolic networks designed to facilitate the mapping of metabolites identified in untargeted metabolomics experiments onto genome-scale metabolic networks. To that end, it streamlines access to known metabolic networks in graph form, and provides this data in a machine-readable, JSON-based format.

Minecraft is an open-world, sandbox building game written in Java, similar in its conception to a virtual Lego construction set, which allows the player to construct arbitrarily large and complex structures, using a variety of visually and even functionally different building blocks. It has been extensively used in educational and scientific contexts in the past, including the illustration of physical, chemical and biological phenomena (Short, 2012). Such applications so far have either taken advantage of ‘naturally’ occurring game events, or necessitated one-off, quasi-manual construction of large-scale structures (Lorch and Mills, 2015). The challenge presented here, given the scale of the putative dataset (i.e. the entire MetExplore database) lies therefore in devising a novel programmatic approach to dynamic, user-driven generation of structures representing arbitrary metabolic networks. We anticipate the use of Metabocraft in teaching biochemistry, at either the secondary school or undergraduate levels, providing students a unique experience of biochemical information.

## 2 Implementation

The MetaboCraft server stack is comprised of three components: a Spigot server, a Shiny server and a plumber server. The Spigot server runs JavaScript code, while the Shiny and plumber servers run R code.

**Fig. 1.**
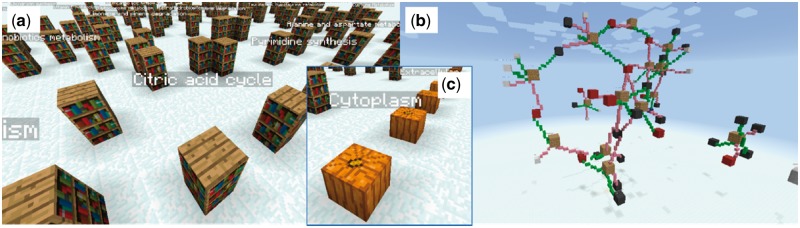
(**a**) Selection of a metabolic pathway via the bookshelves. Maps are created using the Kamada-Kawai algorithm, and a particular pathway may be selected by right-clicking. Subcellular localisation of metabolic maps is also supported. Clicking on a labelled pumpkin (**c**) generates a subset of bookshelves corresponding only to the pathways active in a particular subcellular compartment. (**b**) The citric acid cycle displayed in Minecraft. Glowstones (yellow) represent enzymes, red blocks represent downregulated metabolites, blue blocks represent upregulated ones and black blocks represent unchanged ones. Small white blocks represent side metabolites, such as H_2_O

A series of shell scripts are provided for installing, running and maintenance. The installer automates the downloading and compilation of Spigot, the downloading and installation of the relevant plugins and finally the JavaScript component of MetaboCraft, on top of Spigot. It initializes the configuration of the Minecraft world with a variety of settings appropriate for the desired functionality. It also attempts to automate the installation of the necessary R packages, which due to variations in R setups is not always achievable. The script attempts to anticipate such cases and prompt the user to manually install the missing packages, if necessary. As well as being able to install the components by hand, a Docker image is available that provides a one-click installation option for quick and easy setup.

By default, MetaboCraft displays data from MetExplore BioSource 4324, derived from the Recon 2 reconstruction of the human metabolome (Thiele *et al.*, 2013). Support for other data sources is currently experimental. An example dataset (Stipetic *et al.*, 2016) is provided with the MetaboCraft distribution for demonstration of the uploading feature.

## 3 Results

The Shiny web application can be accessed on port 38909 on the local machine. After optionally uploading their files, on logging into the server using Minecraft, the user is presented with a welcome message informing them of the default viewing parameters and the data files uploaded under their name and available to them. They are presented with a panoramic view of the full default metabolic network map, along with a series of control blocks presenting the choice of cellular compartments. Choosing a compartment presents the user with a view of the network map of that specific compartment, while choosing a pathway from the map presents the 3D graph of that pathway, along with an option to go back to the map view. The various entities represented in a metabolic pathway graph are colour-coded for legibility (Fig. 1).

Several commands are also implemented for the user to input into the Minecraft console, allowing direct access to these features, as well as additional control over the behaviour of MetaboCraft, such as switching between uploaded files and changing the pathway network map layout.

MetaboCraft joins an already crowded field of visualization frameworks for metabolic networks, such as MetExploreViz (Cottret *et al.*, 2010), CytoScape (Shannon, 2003) and TULIP (Auber, 2004). Although it currently lacks the maturity and flexibility of such solutions, it is unique in immersiveness and approachability. Basing the program on a widely recognized 3D modelling platform significantly lowers the barrier to entry for new users with little previous experience of metabolomics, while the Minecraft environment presents a host of as yet untapped features, paving the way for adding elements of real-time interactivity to visualizations in the future.

## Funding

This work was supported by the Wellcome Trust [105614/Z/14/Z].

*Conflict of Interest*: none declared.

## Supplementary Material

Supplementary Data
